# miR-548d-3p Is Up-Regulated in Human Visceral Leishmaniasis and Suppresses Parasite Growth in Macrophages

**DOI:** 10.3389/fcimb.2022.826039

**Published:** 2022-02-10

**Authors:** Eduardo Milton Ramos-Sanchez, Luiza Campos Reis, Marina de Assis Souza, Sandra Márcia Muxel, Kamila Reis Santos, Dimitris Lagos, Valéria Rêgo Alves Pereira, Maria Edileuza Felinto de Brito, Paul Martin Kaye, Lucile Maria Floeter-Winter, Hiro Goto

**Affiliations:** ^1^ Instituto de Medicina Tropical, Faculdade de Medicina, Universidade de São Paulo (IMTSP/USP), São Paulo, Brazil; ^2^ Departamento de Salud Publica, Facultad de Ciencias de La Salud, Universidad Nacional Toribio Rodriguez de Mendoza de Amazonas, Chachapoyas, Peru; ^3^ Graduate Program in Animal Science, Agrarian Sciences Center (CCA), Federal University of Paraiba (UFPB), Areia, Brazil; ^4^ Instituto de Ciências Biomédicas, Universidade de São Paulo, São Paulo, Brazil; ^5^ Veterinary Clinical Immunology Research Group, Departamento de Clínica Médica, Faculdade de Medicina Veterinária e Zootecnia, Universidade de São Paulo, São Paulo, Brazil; ^6^ York Biomedical Research Institute, Hull York Medical School, University of York, York, United Kingdom; ^7^ Instituto Aggeu Magalhães, Fundação Oswaldo Cruz (IAM/FIOCRUZ), Recife, Brazil; ^8^ Instituto de Biociências, Universidade de São Paulo, São Paulo, Brazil; ^9^ Departamento de Medicina Preventiva, Faculdade de Medicina, Universidade de São Paulo, São Paulo, Brazil

**Keywords:** *Leishmania (Leishmania) infantum*, microRNA, visceral leishmaniasis, THP-1 cells, pathogenesis, inflammation

## Abstract

Visceral leishmaniasis caused by *Leishmania (Leishmania) infantum* in Latin America progress with hepatosplenomegaly, pancytopenia, hypergammaglobulinemia, and weight loss and maybe lethal mainly in untreated cases. miRNAs are important regulators of immune and inflammatory gene expression, but their mechanisms of action and their relationship to pathogenesis in leishmaniasis are not well understood. In the present study, we sought to quantify changes in miRNAs associated with immune and inflammatory pathways using the *L. (L.) infantum* promastigote infected- human monocytic THP-1 cell model and plasma from patients with visceral leishmaniasis. We identified differentially expressed miRNAs in infected THP-1 cells compared with non-infected cells using qPCR arrays. These miRNAs were submitted to *in silico* analysis, revealing targets within functional pathways associated with TGF-β, chemokines, glucose metabolism, inflammation, apoptosis, and cell signaling. In parallel, we identified differentially expressed miRNAs in active visceral leishmaniasis patient plasma compared with endemic healthy controls. *In silico* analysis of these data indicated different predicted targets within the TGF-β, TLR4, IGF-I, chemokine, and HIF1α pathways. Only a small number of miRNAs were commonly identified in these two datasets, notably with miR-548d-3p being up-regulated in both conditions. To evaluate the potential biological role of miR-548d-3p, we transiently transfected a miR-548d-3p inhibitor into *L. (L.) infantum* infected-THP-1 cells, finding that inhibition of miR-548d-3p enhanced parasite growth, likely mediated through reduced levels of MCP-1/CCL2 and nitric oxide production. Further work will be required to determine how miR-548d-3p plays a role *in vivo* and whether it serves as a potential biomarker of progressive leishmaniasis.

## Introduction

The leishmaniases are vector-borne diseases caused by protozoan parasites of the order Kinetoplastida, family Trypanosomatidae, and genus *Leishmania*. The leishmaniases are endemic in 98 countries, affecting 0.9 to 1.6 million people globally each year, and are considered the second most important of the neglected diseases caused by protozoa (Bi et al., 2018; Burza et al., 2018; Sasidharan and Saudagar, 2021; WHO, 2021). Transmission occurs when sand flies inject metacyclic promastigotes into the skin. These parasite forms enter phagocytic myeloid cells, transform into amastigotes, and then proliferate, establishing the infection. The diversity of *Leishmania* parasites and vector species and host genetic and immunological conditions lead to different clinical presentations. The three main clinical forms of the disease are cutaneous, mucosal, and visceral leishmaniasis (VL). Human active VL is a severe disease caused by either *Leishmania (Leishmania) donovani* or *L. (L.) infantum* and affects organs rich in mononuclear phagocytes. Active VL manifests with fever, hepatosplenomegaly, pancytopenia, hypergammaglobulinaemia, and significant weight loss and may be lethal mainly in untreated cases (Burza et al., 2018; Ibarra-Meneses et al., 2020; Serafim et al., 2020). Most cases occur in Brazil, East Africa, and India. An estimated 50 000 to 90 000 new cases of VL occur worldwide annually (WHO, 2021).

Data from endemic areas for VL in Brazil indicate that *L. infantum* efficiently induces lifelong protective immunity in most infected individuals that remain asymptomatic (Badaro et al., 1986; Silveira et al., 2009), highlighting the still need to search for factors and mechanisms leading to disease development. Among factors leading to the development of active disease, an immunosuppressive state resulting from either protein-energy malnutrition (Malafaia, 2009) or HIV infection (Lindoso et al., 2014) rank highest. However, participation of other factors gains evidence, including lipoprotein and lipoprotein fraction levels (Carvalho et al., 2014) and growth factors (Reis et al., 2021), and inhibition of secretion of pro-inflammatory cytokines and chemokines (Tasew et al., 2021), along with alterations in the adaptive immune response (Saporito et al., 2013; Palacios et al., 2021).

In active human VL, studies using peripheral blood mononuclear cells (PBMCs) have identified *Leishmania* antigen-specific T cell suppression associated with increased abundance of Transforming growth factor β (TGF-β), interleukin (IL-)10, IL-35 and apoptosis of CD4^+^ T cells. In contrast, studies directly analyzing lymphoid tissues where parasites proliferate, such as bone marrow and spleen, suggest intense immune activation in active VL, including abundant mRNA for tumor necrosis factor α (TNF-α) and interferon γ (IFN-γ) (Goto and Prianti, 2009) and consequently high serum IFN-γ levels (de Medeiros et al., 1998; Samant et al., 2021). Data in the experimental hamster model of VL supports this view, with a broad inflammatory environment found in the spleen that favors parasite proliferation (Kong et al., 2017). This view is consistent with studies showing increased T cell expression of Cytotoxic T-lymphocyte-associated protein 4 (CTLA-4) and Programmed cell death protein 1 (PD-1), indicative of exhausted or anergic T cells (Gautam et al., 2014). The pathogenesis of VL is complex and the factors that cause the disease are not well understood. In this context, many changes in the gene expression profile may occur during infection development, including microRNAs (miRNAs).

miRNAs are a group of small non-coding RNAs (23-25 nucleotides), acting to post-transcriptionally fine-tune gene expression by regulating mRNA levels (Guo et al., 2012; Meng et al., 2012; Liu and Abraham, 2013). miRNAs exert a critical regulatory role in various biological processes, such as cell proliferation, death, reprogramming, signaling, participating in homeostasis, hematopoiesis, cell migration, and regulation of the immune response (Bartel, 2004; Tufekci et al., 2014). In recent years, miRNAs have been shown to play a critical role in developing functional immune responses (Liu and Abraham, 2013). miRNAs have been implicated in various aspects of immunity to viruses, bacteria, and protozoan parasites, including *Trypanosoma*, *Toxoplasma*, *Plasmodium*, and *Leishmania* (Chandan et al., 2019; Acuna et al., 2020; Rashidi et al., 2021). miRNAs have been considered as targets for therapeutic strategies, and since some miRNAs are considered stable in plasma and other biological fluids and are resistant to environmental conditions, they are also attractive as biomarkers of disease or therapeutic response (Sohel, 2016; Takahashi et al., 2019; Souza et al., 2021).

In *Leishmania* infection, modulation of miRNAs expression has been identified in experimental models of visceral and cutaneous leishmaniasis (Acuna et al., 2020; Paul et al., 2020). In VL caused by *L. donovani*, decreased miR-122 expression in the liver of infected mice was associated with an increase in the parasite load (Ghosh et al., 2013). In symptomatic dogs naturally infected with *L. infantum*, expression of miRNAs in PBMCs has been associated with parasite load, with miR-194 and miR-371 positively and miR-150 negatively correlating with the parasitism (Bragato et al., 2018).

In human leishmaniasis, we recently identified a role for miR-548d-3p in cutaneous leishmaniasis caused by *L.* (*Viannia*) *braziliensis*. miR-548d-3p was differentially expressed in plasma from patients with active compared to self-healed cutaneous leishmaniasis and up-regulated in *L. braziliensis*-infected THP-1 cells. A role for miR-548d-3p in directly regulating parasite growth was demonstrated *in vitro*, besides modulation of chemokines MCP1/CCL2, RANTES/CCL5, and IP10/CXCL10 production through miR-548d-3p inhibition (Souza et al., 2021). In another *in vitro* study, more than 300 differentially expressed miRNAs were identified in THP-1 cells infected with an *L. donovani* strain isolated from a patient with post-kala-azar dermal leishmaniasis (Kumar et al., 2020).

In the present study, we analyzed the expression of miRNA related to immune-inflammatory processes during *L. infantum* infection. We studied miRNA abundance in plasma of patients with active VL caused by *L. infantum* compared to healthy non-endemic controls and in THP-1 cells infected *in vitro* with *L. infantum* compared to uninfected THP-1 cells. We observed that differentially expressed miRNAs in these two settings targeted different cellular pathways, including TCR and chemokine signaling and JAK-STAT signaling, as well as cellular processes like apoptosis and peroxisome and phagosome function. miR-548d-3p, previously implicated in *L. braziliensis* infection, was one of the few miRNAs commonly regulated in these two experimental conditions. Transfection of THP-1 cells with a miR-548d-3p inhibitor revealed an effect on inhibiting *Leishmania* growth, likely through induction of MCP-1/CCL2 and nitric oxide production.

## Material And Methods

### Ethics Statement

All individuals agreed to participate by signing the Informed Consent Form. The experimental protocols were approved by the Ethics Committee of the Faculdade de Medicina, Universidade de São Paulo (CAAE 71602217.4.0000.0065) and are in accordance with the World Medical Association Declaration of Helsinki on Ethical Principles for Medical Research lnvolving Human Subjects of 1964, with the latest amendment of 2013. All animal procedures were approved by the Faculdade de Medicina, Universidade de São Paulo, to adhere to institutional guidelines for animal care and use (Protocol n° 336).

### Patients

Individuals were selected from endemic areas for VL in Pernambuco state, Northeastern Brazil. The sample was collected from 8 active VL patients, five males and three females, mean 14 and median 9 years old (1- 48 years old) with confirmed diagnosis by PCR (using the primers RV1 5′-CTTTTCTGGTCCCGCGGGTAGG-3′ and RV2 5′-CCACCTGGCCTATTTTACACCA-3′ targeting the *L. infantum* kinetoplast minicircle DNA) (Ravel et al., 1995). They had no comorbidities such as HIV/AIDS. The control group was six healthy adults recruited from non-endemic areas without previous leishmaniasis or coinfections. Four milliliters of whole blood were collected in EDTA from each individual, and plasma was stored at -80°C until use.

### Parasites

*L. (L.) infantum* (MHOM/BR/72/LD) was maintained through regular passages in the hamster (*Mesocricetus auratus*) to preserve infectivity. Promastigotes were derived from amastigotes purified from the spleen of a hamster and expanded and maintained in 199 medium (Cultilab, Brazil) containing penicillin (100 IU/ml), gentamicin (10 µg/ml), L-glutamine (2mM), HEPES (10 mM) and Hemin (20 mg/ml) and supplemented with 10% heat-inactivated fetal calf serum (FCS) (Cultilab, Brazil) at 26°C. The promastigotes used in the experiments were in the stationary phase of growth and had undergone no more than four passages in culture.

### Infection of Macrophages With *L. infantum*

The THP-1 human monocytic cell line (ATCC) was grown in RPMI 1640 medium (Sigma-Aldrich, USA) supplemented with 2 mM L-glutamine, 1 mM sodium pyruvate, 0.2% sodium bicarbonate, and 5% FCS (complete medium). Cells (5x10^5^) were dispensed onto round 13-mm^2^ glass coverslips in 24-well plates for infectivity analysis, or 2x10^6^ cells in the wells of 24-well plates (Corning Costar, USA) and incubated in the presence of 20 ng/mL phorbol myristate acetate (PMA; Sigma-Aldrich, USA) for 48 hours at 37°C in a humid atmosphere with 5% CO_2_ to allow differentiation into macrophages (Tsuchiya et al., 1982). Then, the wells were washed twice with a culture medium to remove non-adherent cells. Promastigote suspensions (at a multiplicity of infection of 8 parasites per cell) were dispensed into the wells, and infection was allowed to occur for 4 hours at 37°C in a humid atmosphere with 5% CO_2_. After incubation, the non-internalized parasites were washed away, and a complete RPMI medium was added to the wells, beginning the experimental period (0h). The plates were then maintained for 6h or 24h at 37°C in a humid atmosphere with 5% CO_2_. The cells into coverslips were stained for evaluation of parasitism. Cells in 24-well plates were prepared in triplicates per plate, and a pool of cells was considered one biological replicate, and experiment was developed three times to generate three biological replicates for RNA extraction. Uninfected cells were considered as controls.

### Parasite Load in Macrophages

Coverslips were removed from plates, stained with panoptic dyes (Instant Newprov, Brazil), mounted, and processed to evaluate parasitism under a light microscope (Carl Zeiss, Germany); 900 cells per group were counted. The data are presented as the number of parasites per 100 cells [(number of parasites/number of infected cells)x(number of infected cells/total number of cells)x100]. Two independent observers who were blinded to the experimental conditions performed the analysis.

### RNA Extraction, Reverse Transcription and Pre-Amplification

Total RNA extraction from THP-1 cells was performed using the miRVana PARIS isolation kit (Thermo Fisher, USA), and from plasma samples was performed using the miRNeasy Serum/Plasma kit (Qiagen, USA), with the addition of a spike-in control (*Caenorhabditis elegans* cel-miR-39) to ensure the quality of the procedure and to allow qPCR normalization, according to the manufacturer’s instructions. RNA integrity was determined in a spectrophotometer as an OD260/280 absorption ratio between 1.8 and 2.1.

Complementary DNA (cDNA) to template RNA purified from THP-1 cells and plasma samples was synthesized with miScript II RT kit (Qiagen, USA). Briefly, 250 ng of total RNA from THP-1 cells were added to 2 μL of 5X miScript HiSpec Buffer, 1 μL of 10X Nucleics Mix, and 1 μL of miScript Reverse Transcriptase Mix. RNase-free water was added to a final volume of 10 μL. The RNA was incubated for 60 min at 37°C to insert poly-A tail downstream of the miRNA sequence and anneal a T-tail tag for the cDNA elongation. The enzyme was inactivated at 95°C for 5 min. The reaction was performed in the Mastercycler Gradient thermal cycler (Eppendorf, Germany), and the product was stored at −20°C until use. The reverse transcription reaction for plasma samples followed the same protocol, with the manufacturer’s instructions to add 4.5 µL of the purified total RNA. Then, 40 µL of DEPC water was added into each 10 µL RT-PCR product and submitted to a pre-amplification reaction (preAmp), using the miScript PreAmp PCR Kit (Qiagen, USA) according to the manufacturer’s instructions. Then the samples were diluted 10x and stored at -20°C.

### Quantitative Real-Time PCR for miRNA

miRNA expression was evaluated in three biological replicates with the miScript microRNA PCR array (Qiagen, USA), focusing on inflammation and auto-immunity pathway-related molecules (MIHS-105Z). Ready-to-use qPCR plates containing a set of eighty-four specific primers for miRNAs and twelve internal controls were filled in with the previously prepared master mix containing PCR Buffer, SYBR Green, and the 10-fold diluted cDNA for *in vitro* infected THP-1 macrophages or preAmp samples of plasma samples. Quantitative PCR conditions were 40 cycles of 94°C for 15 seconds, 55°C for 30 seconds, and 70°C for 30 seconds (Souza et al., 2021). Normalization of miRNA expression in THP-1-derived macrophages was performed using SNORD95 and RNU6-6p as reference genes amplified in the qPCR plate. The relative expression levels were calculated using the Comparative Ct method, with non-infected cells considered the calibrator group.

For plasma samples, miRNA expression was also evaluated by relative quantification after previous normalization using the miScript miRNAPCR Array Data Analysis software (Qiagen, USA). The cel-miR-39 spike-in control was considered as a technical reference. Simultaneously, a geometric mean of all expressed miRNAs was used as a normalization factor to calculate relative expression to a calibrator group, which varied depending on the analysis.

### *In Silico* miRNA Target Prediction

Target prediction strategy was performed using DIANA-miRpath 3.0 server in the reverse search module (Vlachos et al., 2015), with Targetscan (Agarwal et al., 2015) as the chosen algorithm. To discover potential interactions with other biological pathways related to human leishmaniasis pathogenesis, we performed a second analysis using MiEEA (MiRNA Enrichment Analysis and Annotation), which integrates data from different databases such as miRBase, miRWalk, and miRTarBase (Backes et al., 2016).

### *In Vitro* miRNA Inhibition

The inhibition of miR-548d-3p in THP-1-derived macrophages was performed through a transient transfection protocol. Assays with three different concentrations (3 nM, 10 nM, and 30 nM) of the mirVana^®^ miR-548d3p inhibitor (Ambion, USA) or scrambled Negative Control (Ambion, USA) were performed, and 10 nM concentration was chosen for further use. Before the addition of *L. infantum* promastigotes, a solution containing the miR-548d-3p inhibitor or the negative control together with 3μL of FUGENE transfection reagent (Promega, USA) diluted in 500 uL of RPMI medium previously incubated for 20 min at room temperature was added into each well containing the cells and maintained for 24h (Souza et al., 2021). Simultaneously, non-transfected cells received only a complete RPMI medium. The experiment continued with promastigote infection to evaluate parasitism, chemokine, and nitrite levels in supernatants collected and stored at -80°C until use.

### Evaluation of Chemokine Production

Chemokine quantification was performed using CBA – Human Chemokine Kit (BD Biosciences, USA), following the manufacturer’s instructions in culture supernatants. Briefly, 50 µl of capture beads for MCP1/CCL2, RANTES/CCL5, IL-8/CXCL8, MIG/CXCL9, and IP10/CXCL10, 50 µl of Detection Reagent and 50 µl of the studied sample or standard were added consecutively to each sample tube and incubated for 3 h at room temperature, in the dark. Next, the samples were washed with 1 ml of wash buffer and centrifuged. After discarding the supernatant, the pellet was resuspended in 300 µl buffer and analyzed in a FACS LSR Fortessa flow cytometer (BD Biosciences, USA) (Souza et al., 2021). Raw data were then analyzed using FCAP Array software (BD Biosciences, USA). The detection limits of each chemokine were as follows: 2.7 pg/mL for MCP1/CCL2, 1.0 pg/mL for RANTES/CCL5, 0.2 pg/mL for IL-8/CXCL8, 2.5 pg/mL for MIG/CXCL9 and 2.8 pg/mL for IP10/CXCL10.

### Nitric Oxide (NO) Production

Nitrite (NO_2_) accumulation in the cell culture supernatants was used as an indicator of NO production and was determined using the standard Griess reaction (Green et al., 1982). Fifty microliters of the culture supernatant were reacted with 50 µL of Griess reagent (1% sulfanilamide, 0.1% N-(1-Naphthyl)ethylenediamine dihydrochloride and 2.5% phosphoric acid in bidistilled water) for 10 min at room temperature. The absorbance was measured at 540 nm using a Multiskan MCC/340 P version 2.20 plate reader (Labsystems, Brazil), and the nitrite concentration was calculated using a standard curve for sodium nitrite (NaNO_2_). The tests were run in triplicate.

### Statistical Analysis

Statistical analyses of *in vitro* miRNA expression were performed with Qiagen miScript miRNA PCR Array Data Analysis online software, where data from three independent experiments were submitted to an integrated Student’s t-test under the manufacturer’s recommendation as done in the previous similar work (Muxel et al., 2017). The fold regulation (FR) was considered to be the negative inverse of the fold change [function = −1*(1/fold change value)]. FR ≥ 2 were considered to indicate upregulation, and levels ≤ − 2 were considered to indicate downregulation, as previously described (Muxel et al., 2017). Ex vivo data were also submitted to Student’s t-test, with Bonferroni’s correction, using Microsoft Excel 365. Parasite load data were analyzed by ANOVA with Tukey’s post-test and data from chemokine quantification by Kruskal-Wallis test with Bonferroni’s correction. The differences were considered significant when *P* < 0.05.

## Results

### miRNA Expression in *L. infantum*-Infected THP-1 Cells

**Figure 1:**
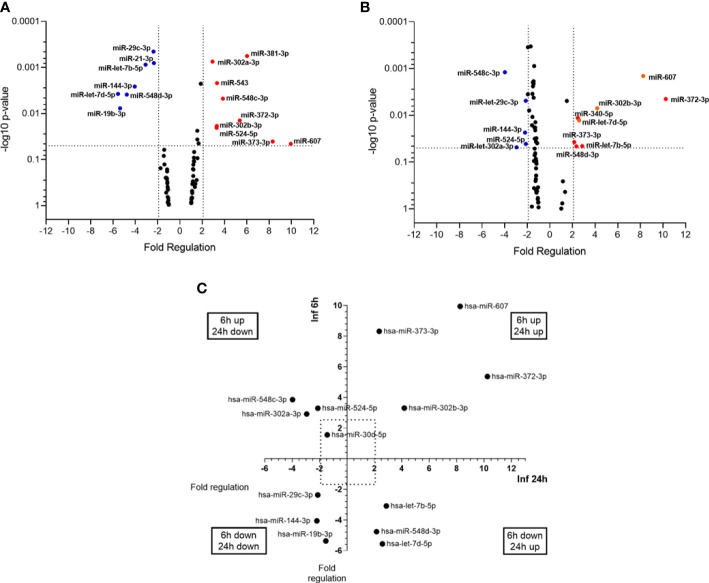
miRNA profiles of *L. (L.) infantum* infected THP-1-macrophages. Volcano plot of differential expression of miRNAs in *L. (L.) infantum* promastigote-infected THP-1 macrophages compared to uninfected macrophages at 6h **(A)** and 24 h **(B)** post-infection. Each dot represents one miRNA. Red dots indicate up-regulated miRNAs, and blue dots represent downregulated ones (P < 0.05). The horizontal black dotted line corresponds to p=0.05, log 10. The relative up- and down-regulation of miRNAs, expressed as boundaries of 2 or -2 of Fold Regulation, respectively. P-value was determined based on a two-tailed Student’s t-test comparing the 6h p.i. or 24 h p.i. with uninfected macrophages. Significantly expressed miRNAs in different times distributed in four groups **(C)**. Results from three biological replicates developed in independent experiments.

miRNA expression was evaluated at 6h and 24h post infection (p.i.), with non-infected THP-1 cells used as a calibrator group. Twenty two (26%) out of 84 miRNAs assayed were significantly altered in expression (P < 0.05) across one or more condition. At 6h p.i., nine miRNAs were upregulated (miR-302a-3p, miR-302b-3p, miR-372-3p, miR-373-3p, miR-381-3p, miR-524-5p, miR-543, miR-548c-3p, miR-607), while seven were down-regulated (let-7b-5p, let-7d-5p, miR-144-3p, miR-19b-3p, miR-21-5p, miR-29c-3p, miR-548d-3p) (**Figure 1A**). At 24h p.i., eight miRNAs were upregulated (let-7b-5p, let-7d-5p, miR-302b-3p, miR-340-5p, miR-372-3p, miR-373-3p, miR-548d-3p, miR-607) and five were downregulated (miR-144-3p, miR-29c-3p miR-302a-3p, miR-524-5p, miR-548c-3p) (**Figure 1B**). Through the time course of infection, we observed that four miRNAs (miR-302b-3p, miR-372-3p, miR-373-3p, miR-607) were upregulated at both 6h and 24h, while miR-144-3p was down-regulated at both times. let-7b-5p, let-7d-5p, miR-340-5p and miR-548d-3p were down-regulated at 6h and upregulated at 24h. In contrast, miR-302a-3p and miR-548c-3p were up-regulated at 6h and down-regulated at 24h (**Figure 1C**). These data indicate that *L. infantum* infection selectively alters miRNA abundance in human macrophage in a time-dependent manner.

### miRNA Expression in Plasma From Active VL Patients

**Figure 2:**
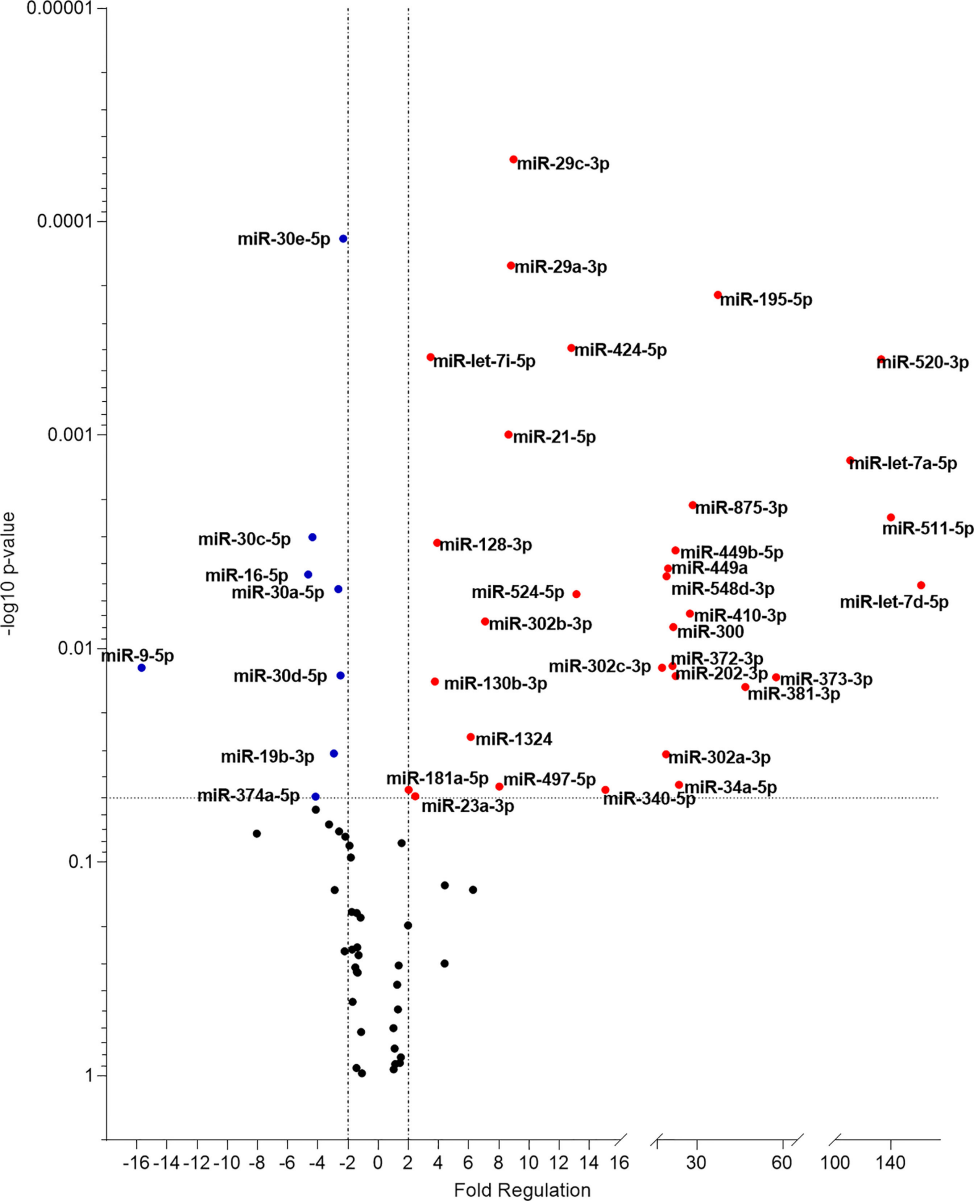
Volcano plot of differential expression of miRNA in plasma from VL patients. Volcano plot showing differential expression of miRNA in plasma samples of active VL patients compared to healthy individuals. Each dot represents one miRNA. Red dots indicate up-regulated miRNAs, and blue dots represent downregulated ones (P < 0.05). The horizontal black dotted line corresponds to p=0.05, log 10. The relative up- and down-regulation of miRNAs, are expressed as boundaries of 2 or -2, of Fold Regulation respectively. P-value was determined based on a two-tailed Student’s t-test. P < 0.05 (Student *t*-test and Bonferroni correction).

miRNA expression was next evaluated in plasma samples from patients with active disease compared to a healthy endemic control group. Forty-two (50%) out of 84 miRNAs assayed were significantly altered in abundance (P < 0.05) (**Figure 2**). 32 miRNAs were up-regulated in patients with active VL, whereas 10 were downregulated (**Table 1**). Of note, 6 miRNAs were commonly up-regulated both in these plasma samples and in infected THP-1 cells (miR-302a-3p, miR-302b-3p, miR-372-3p, miR-373-3p miR-340-5p, and miR-548d-3p) (**Table 1**).

**Table 1:** miRNAs significantly expressed in plasma samples of patients with active disease.

Up-Regulated miRNAs			Down-Regulated miRNAs
hsa-let-7a-5p	hsa-miR-29a-3p	hsa-miR-410-3p	hsa-let-7c-5p
hsa-let-7d-5p	hsa-miR-29c-3p	hsa-miR-424-5p	hsa-miR-101-3p
hsa-let-7i-5p	hsa-miR-300	hsa-miR-449a	hsa-miR-16-5p
hsa-miR-128-3p	**hsa-miR-302a-3p**	hsa-miR-449b-5p	hsa-miR-19b-3p
hsa-miR130b-3p	**hsa-miR-302b-3p**	hsa-miR-497-5p	hsa-miR-30a-5p
hsa-miR-1324	hsa-miR-302c-3p	hsa-miR-511-5p	hsa-miR-30c-5p
hsa-miR-181a-5p	**hsa-miR-340-5p**	hsa-miR-520d-3p	hsa-miR-30d-5p
hsa-miR-195-5p	hsa-miR-34a-5p	hsa-miR-524-5p	hsa-miR-30e-5p
hsa-miR202-3p	**hsa-miR-372-3p**	**hsa-miR-548d-3p**	hsa-miR-374a-5p
hsa-miR-21-5p	**hsa-miR-373-3p**	hsa-miR-875-3p	hsa-miR-9-5p
hsa-miR-23a-3p	hsa-miR-381-3p		

miRNAs in bold, miRNAs up-regulated in infected THP-1 cells and plasma samples of VL patients.

### miRNA Predicted Targets and Their Interactions With Biological Pathways Related to VL

Considering all differentially expressed miRNAs, we searched for target genes and metabolic pathways using Diana MiRPath 3.0 with TargetScan2 as the chosen algorithm to predict miRNA/mRNA interactions. Analyzing modulated miRNAs in infected THP-1 cells, four important metabolic pathways were predicted: the TGF-β pathway with 56 possible target genes; the FOXO transcription factor pathway (Forkhead box) with 74 putative target genes, associated with the control of apoptosis and glucose metabolism; Wnt signaling pathway with 64 putative targets related to foxo, PI3/AKT and the HIF-1 factor pathway with 55 putative target genes associated with anaerobic metabolism in M1 macrophages (**Table S1**).

**Figure 3:**
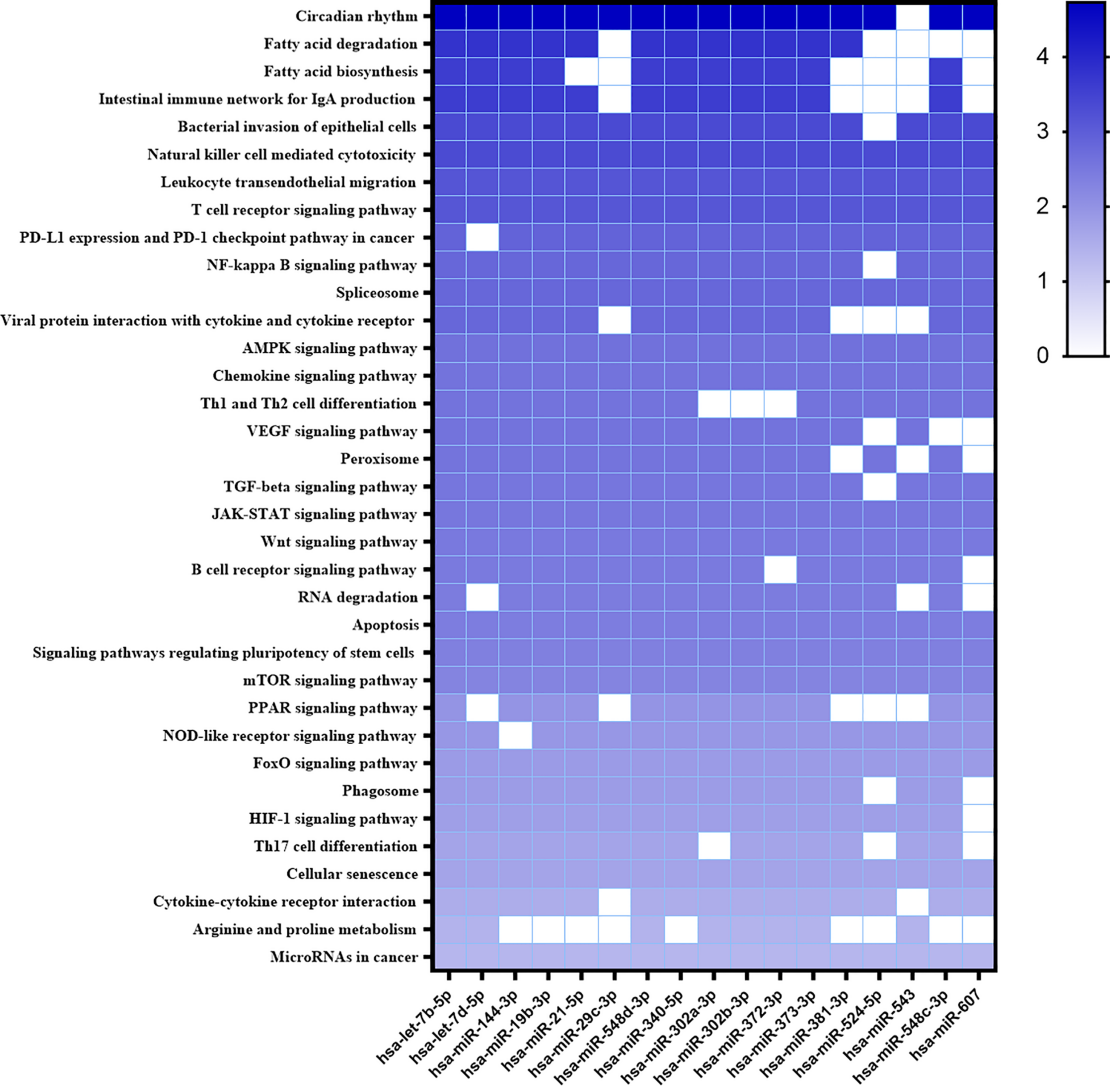
Heatmap of predicted interactions between differentially expressed miRNAs in infected THP-1 cells. The set of differentially expressed microRNAs in *L. infantum*-infected THP-1 at 6 and 24 h and the biological pathways on which they are suggested to act, according to MiEAA algorithms, are shown in heat map format. Results are shown as -log10 of prediction p-value, and the degree of color saturation corresponds to the -log10 of the p-value.

**Figure 4:**
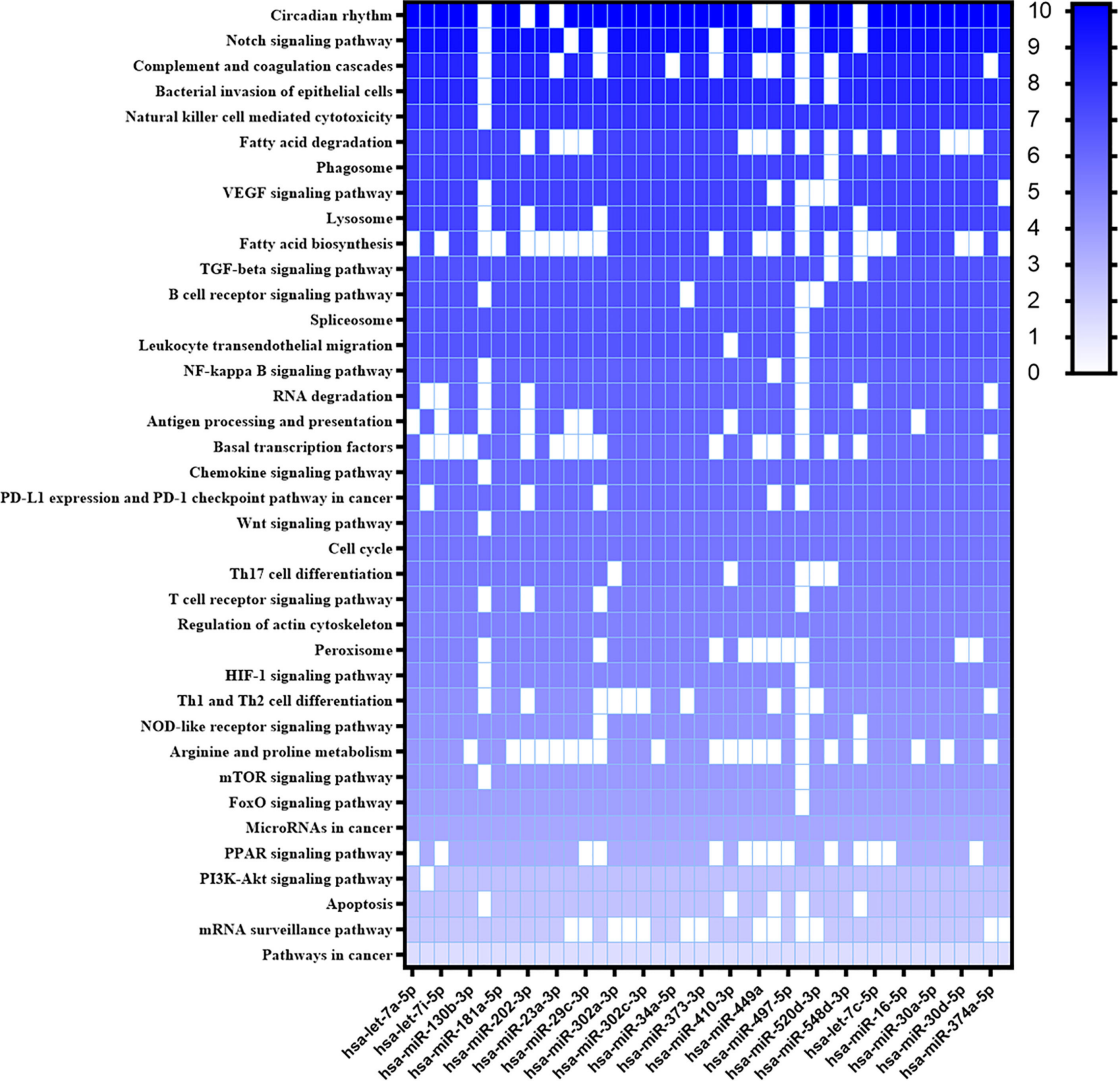
Heatmap of predicted interactions between differentially expressed miRNAs in VL patient plasma. The set of differentially expressed microRNAs in plasma samples from active VL patients caused by *L. infantum* and the biological pathways on which they are suggested to act according to MiEAA algorithms are shown in heat map format. Results are shown as -log10 of prediction p-value, and the degree of color saturation corresponds to the -log10 of the p-value.

New targets were predicted using the MiEEA platform to obtain coverage from more than one classification system (PANTHERDB, WikiPathways, and KEGG). This analysis is worth highlighting those likely targeted by more than one miRNA. There were some pathways known to be important in the *Leishmania*-host interaction like T cell receptor, chemokine signaling pathway, peroxisome, JAK-STAT signaling pathway, apoptosis, PPAR signaling pathway, phagosome, JAK1, MAPK1, PTPN6, PTGS2, of which MAPK1, JAK are involved in the TGFB, FOXO, and HIF1 pathways (**Figure 3**). Some of these pathways have also been observed in our previous study of *L. braziliensis* infection (Souza et al., 2021). Hsa-miR-548d-3p also targets common genes in these pathways. A similar analysis was then conducted with the miRNAs that were found to be differentially regulated in plasma from VL patients (**Figure 4**), revealing a similarly broad and diverse set of potential targets influenced by infection. Among various differentially expressed miRNAs identified, we selected miR-548d-3p, up-regulated in both plasma samples from active VL patients and *L. infantum*-infected THP-1 cells for further validation.

### Effect of miR-548d-3p Inhibition on the Parasite Load in *L. infantum*-Infected THP-1 Cells

**Figure 5:**
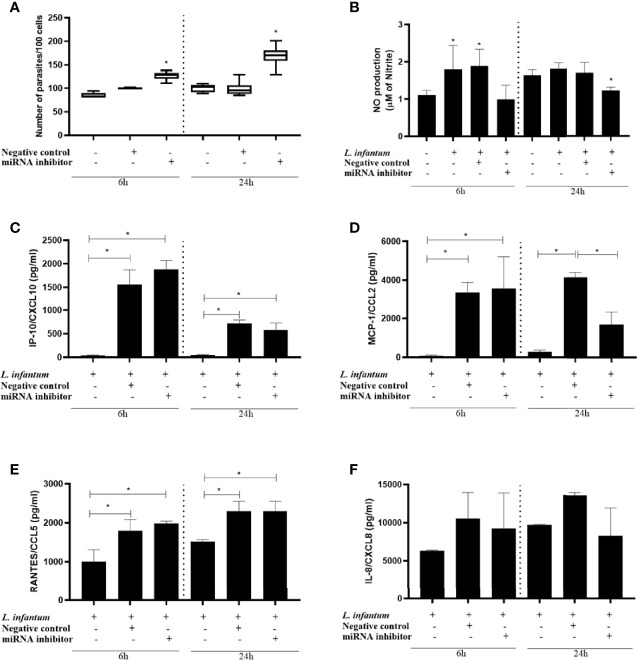
Parasite load, nitric oxide production, and chemokine levels in *L. (L.) infantum* promastigote-infected THP-1 cells transiently transfected with miR-548d-3p inhibitor. THP-1 cells were transiently transfected with miR-548d-3p inhibitor (10 nM) or negative control (scrambled miRNA; 10 nM) as described in Methods. After 24h, macrophages were infected with *L. (L.) infantum* promastigotes and incubated for 6 and 24h post-infection for different evaluations. **(A)** Parasite load represented as the number of parasites/100 cells. **(B)** Nitric oxide (NO) production was determined by the accumulation of nitrite in the cell culture supernatants. IP-10/CXCL10 **(C)**, MCP1/CCL2 **(D)**, RANTES/CXCL5 **(E)**, and IL-8/CXCL8 **(F)** concentrations (pg/mL) were measured by flow cytometry using the CBA kit. * = P < 0.05 (one way ANOVA and Tukey´s test). One representative experiment from three independent assays is shown.

Having identified miR-548d-3p as one of 6 miRNAs affected both in infected THP-1 cells and in plasma from patients with VL, we next sought to identify how this miRNA affected the outcome of infection using the THP-1 model by conducting targeted knockdown experiments. We inhibited miR-548d-3p during *L. infantum* infection using different concentrations (3, 10, and 30 nM) of specific inhibitor or scrambled miRNA. THP-1 cell viability was not affected by the inhibitors at the concentrations used. All concentrations of miR-548d-3p inhibitor increased parasite load in THP-1 cells with the maximal response (1.5 fold increase in parasite numbers) observed at 10 nM and above (**Figure S1**). At both 6h and 24h post-infection, we observed a significant increase in the parasite load when miR-548d-3p was inhibited (P < 0.05), compared with transfection with scrambled RNA (negative control) and non-transfected cells (**Figure 5A**).

### Effect of miR-548d-3p Inhibition on the Nitric Oxide and Chemokine Production in *L. infantum*-Infected THP-1 Cells

As we observed a change in parasite load, we next evaluated NO production, given its role as a major leishmanicidal effector mechanism in macrophages. The effect of miR-548d-3p on nitric oxide (NO) production was assessed at 6 and 24 hours post-infection. We observed a tendency towards decreased NO production at 6h p.i. and a significant decrease at 24h when miR-548d-3p was inhibited (P < 0.05) compared with transfection with scrambled RNA (negative control) and non-transfected cells (**Figure 5B**).

Given that chemokines can regulate and be regulated by NO production and are also stimulated by infection with *Leishmania*, we analyzed the production of chemokines in the supernatant of infected THP-1 cells with or without miR-548d-3p inhibition (**Figures 5C**–**F**). Since the approach was on THP-1 cells, we selected the chemokines related to monocyte/macrophages biology. Production of MCP-1/CCL2, RANTES/CCL5, and IP-10/CXCL10, but not IL-8/CXCL8, was readily observed at 6h p.i. MIG/CXCL9 was undetectable in all samples. Although MCP-1/CCL2 and RANTES/CCL5 expression was maintained at 24h p.i., MIG/CXCL10 expression was reduced at this later time, possibly reflecting limited induction of interferon in this system. Amongst these four chemokines, only the production of MCP-1/CCL2 (**Figure 5D**) was significantly decreased by miRNA inhibition and only at 24 h p.i. Although NO has been shown to be positively regulated by MCP-1/CCL2 (Biswas et al., 2001), the more immediate impact of miR-548d-3p inhibition on NO production (**Figure 5A**) suggests that this may occur independently of its effect on MCP-1/CCL2 production.

### Chemokine Levels in the Plasma of VL Patients

**Figure 6:**
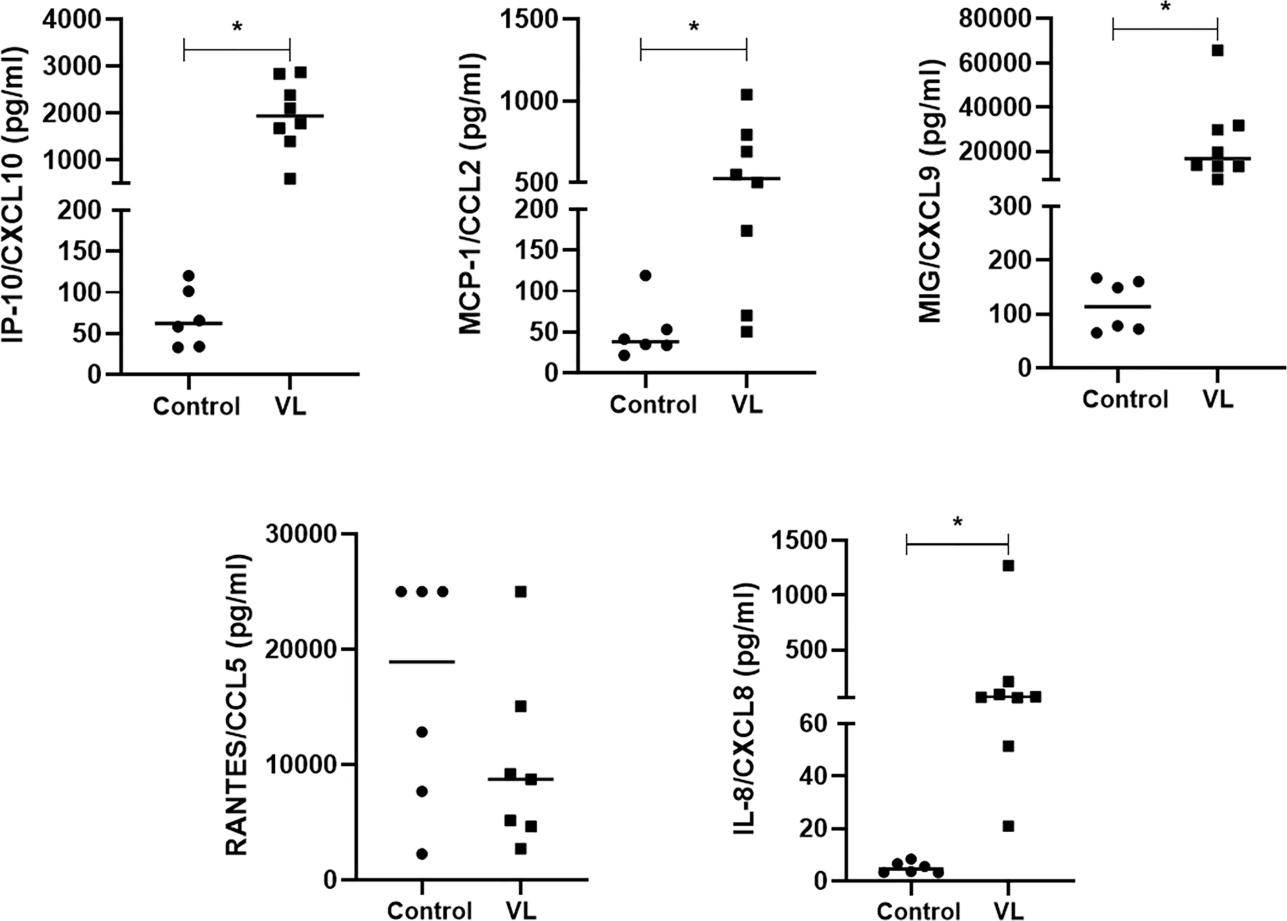
Chemokines in plasma of active VL patients and controls. IP-10/CXCL10, MCP1/CCL2, MIG/CXCL9, RANTES/CXCL5, and IL-8/CXCL8 concentrations were measured in the CBA kit using flow-cytometry. Data are shown as pg/mL, and each symbol corresponds to one subject * = p <0.05 (Mann-Whitney test). Patients (N=8) and controls (N=6).

Finally, we examined the plasma of VL patients and controls for expression of the same set of chemokines. VL patients showed significantly increased IP-10/CXCL10, MCP-1/CCL2, MIG/CXCL9, and IL-8/CXCL8 chemokines compared to the control group (healthy individuals). No difference was observed in RANTES/CXCL5 production (**Figure 6**).

## Discussion

In the present study, we examined miRNA expression in *L. infantum* infection to identify signatures likely associated with the pathogenic process. Few studies have evaluated the participation of miRNAs during *Leishmania* infection (Lemaire et al., 2013; Muxel et al., 2017; Muxel et al., 2018; Fernandes et al., 2019; Paul et al., 2020; Rashidi et al., 2021; Souza et al., 2021). We compared miRNAs in plasma of patients with VL caused by *L. infantum* and in human monocyte-derived THP-1 cells infected *in vitro* with *L. infantum*.

Our data are the first to report on the profile of miRNAs in plasma from patients with active VL caused by *L. infantum*. We identified 32 miRNAs up-regulated and 10 down-regulated in plasma during VL infection compared to plasma-derived from healthy endemic controls. Amongst up-regulated miRNAs, six were also up-regulated *in vitro* in THP-1 cells, namely miR-302a-3p, miR-302b-3p, miR-340-5p, miR-372-3p, miR-373-3p, and miR-548d-3p, suggesting that these miRNAs can be directly regulated in macrophages as a consequence of intracellular parasitism. One of these, miR-548d-3p, had not previously been described with *L. infantum* infection. Therefore, we determined whether it played a functional role during *in vitro* infection.

The miR-548 family, only described in primates, comprises over 69 identified miRNAs present on almost all human chromosomes and is one of the larger yet more poorly conserved microRNA families. The miR-548 family is implicated in different biological processes, including regulating important signaling pathways: MAPK, Phosphatidylinositol (PI3K), p53, B cell receptor, T cell receptor, TGF-beta, PPAR, Calcium, and Insulin signaling pathways. It also has a role in human tumorigenesis (Liang et al., 2012).

Members of this miRNA family have been shown to target immune system-associated genes and have been described as differentially expressed in other diseases. miR-548ah-5p is up-regulated at the immune activation phase of chronic hepatitis B patients, and *in vitro* studies using THP-1 cells treated with miR-548ah-5p mimics suggest that it may involve the reduced expression of IFN-γR1, which may, in turn, facilitate viral pathogenesis (Xing et al., 2014). Other members of the miR-548 family down-regulate host antiviral response *via* direct targeting interferon-λ1 (IFN-λ1). For example, inhibition of miR-548b-5p, miR-548c-5p, miR-548i, and miR-548j increased IFN-λ1 secretion, and the targeted IFN-λ1 gene can induce IP-10, MCP1, IL-8 (Fu et al., 2021). The chemokine MCP-1 was seen affected by miR548d-3p in the present study. Due to the diversity of signaling pathways regulated by the miR-548 family and its participation in *Leishmania* infection in our previous results in *L. braziliensis* infection affecting parasite growth and chemokine production (Souza et al., 2021), we proceeded to evaluate the role of miR-548d-3p in *L. infantum* infection.

miR-548d-3p was up-regulated in plasma from VL patients and in *L. infantum* infected- THP-1 cells *in vitro*. Our results also show that *L. infantum* infected-THP-1 cells treated with miR-548d-3p inhibitor had increased parasite load and decreased expression of MCP1 and NO. MCP-1 is known to induce the production and release of NO, a leishmanicidal molecule, explaining in part the increase in parasitism in the presence of the miRNA inhibitor. However, the MCP-1 level in serum samples was elevated in active VL that presents with high parasitism. However, in another study, it was even higher after 6 months of treatment and in asymptomatic patients (Ibarra-Meneses et al., 2017). These data suggest that MCP-1 affects parasite growth inhibition but not as a single anti-leishmanial element. In addition, full concordance across *in vitro* and *in vivo* data would not be expected as multiple cells, and tissue sources may contribute to MCP1 plasma levels *in vivo*, many of which would not be directly subject to intracellular parasitism. In our previous study, miR-548d-3p was up-regulated in plasma from self-healed cutaneous leishmaniasis patients and *in vitro L. braziliensis*-infected THP-1 cells, and when miR-548d-3p was inhibited in infected THP-1 cells, a decrease in parasite load was observed, and an increase in the production of MCP1/CCL2, RANTES/CCL5, and IP-10/CXCL10 (Souza et al., 2021). These differences may be due to species-specific mechanisms for immune system activation and evasion and the differences (cutaneous or systemic) in clinical presentation. Other members of the 548-miRNA family, miR-548at-5p and miR-548t-3p, were up-regulated in THP-1 cells infected with promastigotes isolated from VL and post kala-azar dermal leishmaniasis patients caused by *L. donovani*. These were seen as related to inflammation and apoptosis (Kumar et al., 2020).

It was observed that MCP-1 cytokine mRNA is more abundant in lesions of *L. mexicana*-infected patients who developed self-healing localized lesions when compared to lesions from chronic diffuse cutaneous leishmaniasis. This finding suggests that the cytokine MCP-1 may contribute to the spontaneous healing process (Ritter et al., 1996). Using human monocytes infected with *L. major*, it was demonstrated that MCP1 chemokine activates monocytes and decreases parasitism, possibly by producing intermediate reactive oxygen species (Ritter and Moll, 2000). In the same line, human peripheral macrophages infected with *L. infantum* showed that MCP-1 enhances macrophage’s nitric oxide production and release at levels induced by INF-γ stimulation. This NO production results in decreasing parasitism (Brandonisio et al., 2002).

Another study demonstrated that *L. infantum*-infected whole blood cells from asymptomatic patients, when stimulated with soluble *Leishmania* antigen, produce significantly higher levels of MCP1 than patients with active or cured VL. Whole blood cells from cured patients have significantly higher levels of MCP-1 after 6 months of the treatment (Ibarra-Meneses et al., 2017). These data reinforce that MCP-1 is essential for a protective immune response and can be used as a biomarker for a protective immune response against *L. infantum.* The increase in parasitism observed in the present study may suggest inhibition of NO production *via* MCP1. However, other studies showed the activation of PI3K/AKT signaling pathway activation upon knockdown of miR-548b-3p with further suppression of cell apoptosis and enhancement of cell proliferation (Xie et al., 2017; Wang et al., 2020). The increase the parasitism in the presence of the miRNA inhibition *in vitro* may also be related to the activation of the PI3K/AKT signaling pathway.

Our ex-vivo results show that patients with active VL have significantly higher IP10/CXCL10, MIG/CXCL9, IL-8/CXCL8, and MCP1/CCL2 compared with the control group, probably an attempt of the immune system to control *Leishmania* infection. IP-10 seems to be related to the control of *L. amazonensis* infection and also protects against VL (Vasquez and Soong, 2006; Fallahi et al., 2016). In the active VL, if miR-548d-3p is acting to control the parasite growth, it should have other elements counteracting to maintain infection progression seen in these cases.

In addition to miR-548d-3p, other miRNAs identified in this study have also been observed to change expression in studies of *Leishmania* infection. These miRNAs presented below in sequence are related to activation or modulation of the immune system and may play a crucial role in *Leishmania* infection either in the control or progression of the infection. The following miRNAs may contribute to the progression of the infection: a) miR-21 seen up-regulated in dendritic cells, macrophages, inflammatory monocytes, polymorphonuclear neutrophils, and in the spleen and liver tissues after *L. donovani* infection and in splenic leukocytes from *L. infantum*-naturally infected dogs and related to the inhibition of IL-12 expression and impairment of the Th1 response implied in the control of the infection (Melo et al., 2019; Varikuti et al., 2021); b) miR-302a-3p that regulates receptor activator of nuclear factor kappa-B ligand (RANKL) (a member of the tumor necrosis factor TNF superfamily) that regulates T-cell-dependent immune response and apoptosis (Irwandi et al., 2018) and also up-regulated in *in vitro L. braziliensis* infection (Souza et al., 2021); c) miR-372-3p that inhibits cell proliferation and apoptosis and seen up-regulated in *L. braziliensis* infection (Chen et al., 2015; Souza et al., 2021). Otherwise, distinct miRNAs may act on the control of the infection: a) miR-340-5p that targets the IL-4, major Th2 cytokines secreted by CD4^+^ T cells, and this miRNA, different from observed with *L. infantum*, was down-regulated in *in vitro L. donovani* infection (Kumar et al., 2020); b) miR-302b-3p that inhibits cell proliferation through the AKT pathway by targeting insulin-like growth factor 1 receptor (IGF-1R) (Guo et al., 2017) and was also up-regulated in *in vitro L. amazonensis* and *L. braziliensis* infection (Muxel et al., 2017; Souza et al., 2021); c) miR-373-3p that is implicated in the regulation of cell proliferation, apoptosis, senescence, migration, and cell invasion and was up-regulated in *L. braziliensis* infection (Wei et al., 2015; Souza et al., 2021).

Our in silico results show that up-regulated microRNAs in plasma and THP-1 cells can target processes or pathways of TGF-β signaling, T cell receptor signaling chemokine signaling, cytokine-cytokine receptor interaction, NOD-like receptor signaling, arginine a proline metabolism. From the total up-regulated microRNAs seen in the present study, six miRNAs are also up-regulated in plasma and THP-1 samples and target important pathways. When inhibited, phagosome and peroxisome processes may result in a favorable microenvironment for amastigote parasites, including the inhibition of apoptosis by infection. Other pathways like the NOD pathway associated with inflammasome, TGF-β signaling pathway, INHBA gene that may modulate MCP-1, IP-10, NO production, arginine, proline metabolism, chemokine signaling were targeted. These pathways are involved directly or indirectly in chemokine, cytokine signaling, or gene expression, including MCP-1 and NO production altered or modulated in the present experiments upon miR-548d-3p inhibition.

In conclusion, this study of VL patients and using *L. infantum*-infected THP-1 cells extend previous observations on the role of miR-548d-3p in cutaneous leishmaniasis caused by *L. braziliensis* (Souza et al., 2021). Our data open new possibilities to study the interactions of the 548 family and the respective targeted genes, using the miR-548d-3p as a tool directed to the regulation of immune or pathological processes in humans/primates leishmaniases. In addition, miR-548d-3p may be explored as a biomarker of prognostic value, and targeting miR-548d-3p may provide new therapeutic opportunities in leishmaniasis and other inflammatory diseases.

## Data Availability Statement

The original contributions presented in the study are included in the article/**Supplementary Material**. Further inquiries can be directed to the corresponding author.

## Ethics Statement

The studies involving human participants were reviewed and approved by Ethics Committee of the Faculdade de Medicina, Universidade de São Paulo (CAAE 71602217.4.0000.0065). The patients/participants provided their written informed consent to participate in this study. All animal procedures were approved by the Faculdade de Medicina, Universidade de São Paulo to adhere to institutional guidelines for animal care and use (Protocol n° 336).

## Author Contributions

HG: Conceptualization, study design, project and researcher supervision, data analysis, and manuscript preparation. ER-S, LR, and MS: Conceptualization, study design, experimental work, data analysis, manuscript preparation. SM: study design, experimental work, data analysis, manuscript preparation. KS: experimental work; VP and MB: coordination of sample and data collection in the endemic area; data interpretation. LF-W: study design, researcher supervision, data analysis, and manuscript preparation. DL and PK: data analysis, manuscript preparation. All authors contributed to the article and approved the submitted version.

## Funding

This work was supported by the Fundação de Amparo à Pesquisa do Estado de São Paulo (grants 2018/23512-0, 2018/14398-0 and 2018/24693-9, fellowship 2014/14756-2 to MS and 2019/25393-1 to LR), Medical Research Council (grants MR/P024661/1 and MR/S019472), the Conselho Nacional de Pesquisa (research fellowship to HG), the Coordenação de Aperfeiçoamento de Pessoal de Nível Superior (CAPES; fellowship to MS) and LIM 38 (Hospital das Clínicas, Faculdade de Medicina, Universidade de São Paulo).

## Conflict of Interest

The authors declare that the research was conducted in the absence of any commercial or financial relationships that could be construed as a potential conflict of interest.

## Publisher’s Note

All claims expressed in this article are solely those of the authors and do not necessarily represent those of their affiliated organizations, or those of the publisher, the editors and the reviewers. Any product that may be evaluated in this article, or claim that may be made by its manufacturer, is not guaranteed or endorsed by the publisher.

## Supplementary Material

Supplementary Figure 1 Parasite load (number of parasites/100 cells) in *L. (L.) infantum* promastigote-infected THP-1 cells transiently transfected with miR-548d-3p inhibitor. THP-1 cells were transiently transfected with miR-548d-3p inhibitor (miR = 3, 10 and 30 nM) or negative control (NC - scrambled miRNA = 3, 10 and 30 nM) as described in Methods. After 24h, cells were infected with *L. (L.) infantum* promastigotes, and parasite load in macrophages was assessed at 6 hours post-infection **(A)** and 24 hours post-infection **(B)**. * = P < 0.05 (one-way ANOVA and Tukey´s test) compared to the control groups. One representative experiment from three independent assays is shown.

## References

[B1] Acuna S. M. Floeter-Winter L. M. Muxel S. M. (2020). MicroRNAs: Biological Regulators in Pathogen-Host Interactions. Cells 9 (1), 113. doi: 10.3390/cells9010113 PMC7016591 31906500

[B2] Agarwal V. Bell G. W. Nam J. W. Bartel D. P. (2015). Predicting Effective microRNA Target Sites in Mammalian mRNAs. Elife 4, e05005. doi: 10.7554/eLife.05005 PMC4532895 26267216

[B3] Backes C. Khaleeq Q. T. Meese E. Keller A. (2016). miEAA: microRNA Enrichment Analysis and Annotation. Nucleic Acids Res. 44 (W1), W110–W116. doi: 10.1093/nar/gkw345 27131362 PMC4987907

[B4] Badaro R. Jones T. C. Lorenco R. Cerf B. J. Sampaio D. Carvalho E. M.. (1986). A Prospective Study of Visceral Leishmaniasis in an Endemic Area of Brazil. J. Infect. Dis. 154 (4), 639–649. doi: 10.1093/infdis/154.4.639 3745974

[B5] Bartel D. P. (2004). MicroRNAs: Genomics, Biogenesis, Mechanism, and Function. Cell 116 (2), 281–297. doi: 10.1016/s0092-8674(04)00045-5 14744438

[B6] Bi K. Chen Y. Zhao S. Kuang Y. John Wu C. H. (2018). Current Visceral Leishmaniasis Research: A Research Review to Inspire Future Study. BioMed. Res. Int. 2018, 9872095. doi: 10.1155/2018/9872095 30105272 PMC6076917

[B7] Biswas S. K. Sodhi A. Paul S. (2001). Regulation of Nitric Oxide Production by Murine Peritoneal Macrophages Treated *In Vitro* With Chemokine Monocyte Chemoattractant Protein 1. Nitric Oxide 5 (6), 566–579. doi: 10.1006/niox.2001.0370 11730364

[B8] Bragato J. P. Melo L. M. Venturin G. L. Rebech G. T. Garcia L. E. Lopes F. L.. (2018). Relationship of Peripheral Blood Mononuclear Cells miRNA Expression and Parasitic Load in Canine Visceral Leishmaniasis. PloS One 13 (12), e0206876. doi: 10.1371/journal.pone.0206876 30517108 PMC6281177

[B9] Brandonisio O. Panaro M. A. Fumarola I. Sisto M. Leogrande D. Acquafredda A.. (2002). Macrophage Chemotactic Protein-1 and Macrophage Inflammatory Protein-1 Alpha Induce Nitric Oxide Release and Enhance Parasite Killing in Leishmania Infantum-Infected Human Macrophages. Clin. Exp. Med. 2 (3), 125–129. doi: 10.1007/s102380200017 12447609

[B10] Burza S. Croft S. L. Boelaert M. (2018). Leishmaniasis. Lancet 392 (10151), 951–970. doi: 10.1016/S0140-6736(18)31204-2 30126638

[B11] Carvalho M. D. Alonso D. P. Vendrame C. M. Costa D. L. Costa C. H. Werneck G. L.. (2014). Lipoprotein Lipase and PPAR Alpha Gene Polymorphisms, Increased Very-Low-Density Lipoprotein Levels, and Decreased High-Density Lipoprotein Levels as Risk Markers for the Development of Visceral Leishmaniasis by Leishmania Infantum. Mediators Inflamm. 2014, 230129. doi: 10.1155/2014/230129 25242866 PMC4163308

[B12] Chandan K. Gupta M. Sarwat M. (2019). Role of Host and Pathogen-Derived MicroRNAs in Immune Regulation During Infectious and Inflammatory Diseases. Front. Immunol. 10, 3081. doi: 10.3389/fimmu.2019.03081 32038627 PMC6992578

[B13] Chen X. Hao B. Han G. Liu Y. Dai D. Li Y.. (2015). miR-372 Regulates Glioma Cell Proliferation and Invasion by Directly Targeting PHLPP2. J. Cell Biochem. 116 (2), 225–232. doi: 10.1002/jcb.24949 25160587

[B14] de Medeiros I. M. Castelo A. Salomao R. (1998). Presence of Circulating Levels of Interferon-Gamma, Interleukin-10 and Tumor Necrosis Factor-Alpha in Patients With Visceral Leishmaniasis. Rev. Inst Med. Trop. Sao Paulo 40 (1), 31–34. doi: 10.1590/s0036-46651998000100007 9713135

[B15] Fallahi P. Elia G. Bonatti A. (2016). Leishmaniasis and IFN-Gamma Dependent Chemokines. Clin. Ter 167 (5), e117–e122. doi: 10.7417/CT.2016.1954 27845489

[B16] Fernandes J. C. R. Aoki J. I. Maia Acuna S. Zampieri R. A. Markus R. P. Floeter-Winter L. M.. (2019). Melatonin and Leishmania Amazonensis Infection Altered miR-294, miR-30e, and miR-302d Impacting on Tnf, Mcp-1, and Nos2 Expression. Front. Cell. Infect. Microbiol. 9, 60. doi: 10.3389/fcimb.2019.00060 30949455 PMC6435487

[B17] Fu L. X. Chen T. Guo Z. P. Cao N. Zhang L. W. Zhou P. M. (2021). Enhanced Serum Interferon-Lambda 1 Interleukin-29 Levels in Patients With Psoriasis Vulgaris. Bras. Dermatol. 96 (4), 416–421. doi: 10.1016/j.abd.2020.11.007 PMC8245709 34030913

[B18] Gautam S. Kumar R. Singh N. Singh A. K. Rai M. Sacks D.. (2014). CD8 T Cell Exhaustion in Human Visceral Leishmaniasis. J. Infect. Dis. 209 (2), 290–299. doi: 10.1093/infdis/jit401 23922369 PMC3873784

[B19] Ghosh J. Bose M. Roy S. Bhattacharyya S. N. (2013). Leishmania Donovani Targets Dicer1 to Downregulate miR-122, Lower Serum Cholesterol, and Facilitate Murine Liver Infection. Cell Host Microbe 13 (3), 277–288. doi: 10.1016/j.chom.2013.02.005 23498953 PMC3605572

[B20] Goto H. Prianti M. (2009). Immunoactivation and Immunopathogeny During Active Visceral Leishmaniasis. Rev. Inst Med. Trop. Sao Paulo 51 (5), 241–246. doi: 10.1590/s0036-46652009000500002 19893975

[B21] Green L. C. Wagner D. A. Glogowski J. Skipper P. L. Wishnok J. S. Tannenbaum S. R. (1982). Analysis of Nitrate, Nitrite, and [15N]Nitrate in Biological Fluids. Anal. Biochem. 126 (1), 131–138. doi: 10.1016/0003-2697(82)90118-X 7181105

[B22] Guo Z. Shao L. Zheng L. Du Q. Li P. John B.. (2012). miRNA-939 Regulates Human Inducible Nitric Oxide Synthase Posttranscriptional Gene Expression in Human Hepatocytes. Proc. Natl. Acad. Sci. U. S. A. 109 (15), 5826–5831. doi: 10.1073/pnas.1118118109 22451906 PMC3326458

[B23] Guo B. Zhao Z. Wang Z. Li Q. Wang X. Wang W.. (2017). MicroRNA-302b-3p Suppresses Cell Proliferation Through AKT Pathway by Targeting IGF-1R in Human Gastric Cancer. Cell Physiol. Biochem. 42 (4), 1701–1711. doi: 10.1159/000479419 28743112

[B24] Ibarra-Meneses A. V. Moreno J. Carrillo E. (2020). New Strategies and Biomarkers for the Control of Visceral Leishmaniasis. Trends Parasitol. 36 (1), 29–38. doi: 10.1016/j.pt.2019.10.005 31718888

[B25] Ibarra-Meneses A. V. Sanchez C. Alvar J. Moreno J. Carrillo E. (2017). Monocyte Chemotactic Protein 1 in Plasma From Soluble Leishmania Antigen-Stimulated Whole Blood as a Potential Biomarker of the Cellular Immune Response to Leishmania Infantum. Front. Immunol. 8, 1208. doi: 10.3389/fimmu.2017.01208 29033933 PMC5626820

[B26] Irwandi R. A. Khonsuphap P. Limlawan P. Vacharaksa A. (2018). miR-302a-3p Regulates RANKL Expression in Human Mandibular Osteoblast-Like Cells. J. Cell Biochem. 119 (6), 4372–4381. doi: 10.1002/jcb.26456 29058810

[B27] Kong F. Saldarriaga O. A. Spratt H. Osorio E. Y. Travi B. L. Luxon B. A.. (2017). Transcriptional Profiling in Experimental Visceral Leishmaniasis Reveals a Broad Splenic Inflammatory Environment That Conditions Macrophages Toward a Disease-Promoting Phenotype. PloS Pathog. 13 (1), e1006165. doi: 10.1371/journal.ppat.1006165 28141856 PMC5283737

[B28] Kumar A. Vijaykumar S. Dikhit M. R. Abhishek K. Mukherjee R. Sen A.. (2020). Differential Regulation of miRNA Profiles of Human Cells Experimentally Infected by Leishmania Donovani Isolated From Indian Visceral Leishmaniasis and Post-Kala-Azar Dermal Leishmaniasis. Front. Microbiol. 11, 1716. doi: 10.3389/fmicb.2020.01716 32849363 PMC7410929

[B29] Lemaire J. Mkannez G. Guerfali F. Z. Gustin C. Attia H. Sghaier R. M.. (2013). MicroRNA Expression Profile in Human Macrophages in Response to Leishmania Major Infection. PloS Negl. Trop. Dis. 7 (10), e2478. doi: 10.1371/journal.pntd.0002478 24098824 PMC3789763

[B30] Liang T. Guo L. Liu C. (2012). Genome-Wide Analysis of Mir-548 Gene Family Reveals Evolutionary and Functional Implications. J. BioMed. Biotechnol. 2012, 679563. doi: 10.1155/2012/679563 23091353 PMC3468316

[B31] Lindoso J. A. Cota G. F. da Cruz A. M. Goto H. Maia-Elkhoury A. N. Romero G. A.. (2014). Visceral Leishmaniasis and HIV Coinfection in Latin America. PloS Negl. Trop. Dis. 8 (9), e3136. doi: 10.1371/journal.pntd.0003136 25233461 PMC4169383

[B32] Liu G. Abraham E. (2013). MicroRNAs in Immune Response and Macrophage Polarization. Arterioscler. Thromb. Vasc. Biol. 33 (2), 170–177. doi: 10.1161/ATVBAHA.112.300068 23325473 PMC3549532

[B33] Malafaia G. (2009). Protein-Energy Malnutrition as a Risk Factor for Visceral Leishmaniasis: A Review. Parasite Immunol. 31 (10), 587–596. doi: 10.1111/j.1365-3024.2009.01117.x 19751470

[B34] Melo L. M. Bragato J. P. Venturin G. L. Rebech G. T. Costa S. F. Garcia L. E.. (2019). Induction of miR 21 Impairs the Anti-Leishmania Response Through Inhibition of IL-12 in Canine Splenic Leukocytes. PloS One 14 (12), e0226192. doi: 10.1371/journal.pone.0226192 31825987 PMC6905561

[B35] Meng S. Cao J. T. Zhang B. Zhou Q. Shen C. X. Wang C. Q. (2012). Downregulation of microRNA-126 in Endothelial Progenitor Cells From Diabetes Patients, Impairs Their Functional Properties, *via* Target Gene Spred-1. J. Mol. Cell Cardiol. 53 (1), 64–72. doi: 10.1016/j.yjmcc.2012.04.003 22525256

[B36] Muxel S. M. Acuña S. M. Aoki J. I. Zampieri R. A. Floeter-Winter L. M. (2018). Toll-Like Receptor and miRNA-Let-7e Expression Alter the Inflammatory Response in Leishmania Amazonensis-Infected Macrophages. Front. Immunol. 9, 2792. doi: 10.3389/fimmu.2018.02792 30555476 PMC6283264

[B37] Muxel S. M. Laranjeira-Silva M. F. Zampieri R. A. Floeter-Winter L. M. (2017). Leishmania (Leishmania) Amazonensis Induces Macrophage miR-294 and miR-721 Expression and Modulates Infection by Targeting NOS2 and L-Arginine Metabolism. Sci. Rep. 7, 44141. doi: 10.1038/srep44141 28276497 PMC5343489

[B38] Palacios G. Diaz-Solano R. Valladares B. Dorta-Guerra R. Carmelo E. (2021). Early Transcriptional Liver Signatures in Experimental Visceral Leishmaniasis. Int. J. Mol. Sci. 22 (13), 7161. doi: 10.3390/ijms22137161 34281214 PMC8267970

[B39] Paul S. Ruiz-Manriquez L. M. Serrano-Cano F. I. Estrada-Meza C. Solorio-Diaz K. A. Srivastava A. (2020). Human microRNAs in Host-Parasite Interaction: A Review. 3 Biotech. 10 (12), 510. doi: 10.1007/s13205-020-02498-6 PMC7644590 33178551

[B40] Rashidi S. Mansouri R. Ali-Hassanzadeh M. Ghani E. Barazesh A. Karimazar M.. (2021). Highlighting the Interplay of microRNAs From Leishmania Parasites and Infected-Host Cells. Parasitology 148 (12), 1434–1446. doi: 10.1017/S0031182021001177 34218829 PMC11010138

[B41] Ravel S. Cuny G. Reynes J. Veas F. (1995). A Highly Sensitive and Rapid Procedure for Direct PCR Detection of Leishmania Infantum Within Human Peripheral Blood Mononuclear Cells. Acta Tropica 59 (3), 10. doi: 10.1016/0001-706X(95)00079-T 7572425

[B42] Reis L. C. Ramos-Sanchez E. M. Araujo F. N. Leal A. F. Ozaki C. Y. Sevillano O. R.. (2021). Pleiotropic Effect of Hormone Insulin-Like Growth Factor-I in Immune Response and Pathogenesis in Leishmaniases. J. Immunol. Res. 2021, 6614475. doi: 10.1155/2021/6614475 34036108 PMC8116165

[B43] Ritter U. Moll H. (2000). Monocyte Chemotactic Protein-1 Stimulates the Killing of Leishmania Major by Human Monocytes, Acts Synergistically With IFN-Gamma and Is Antagonized by IL-4. Eur. J. Immunol. 30 (11), 3111–3120. doi: 10.1002/1521-4141(200011)30:11<3111::AID-IMMU3111>3.0.CO;2-O 11093125

[B44] Ritter U. Moll H. Laskay T. Brocker E. Velazco O. Becker I.. (1996). Differential Expression of Chemokines in Patients With Localized and Diffuse Cutaneous American Leishmaniasis. J. Infect. Dis. 173 (3), 699–709. doi: 10.1093/infdis/173.3.699 8627035

[B45] Samant M. Sahu U. Pandey S. C. Khare P. (2021). Role of Cytokines in Experimental and Human Visceral Leishmaniasis. Front. Cell. Infect. Microbiol. 11, 624009. doi: 10.3389/fcimb.2021.624009 33680991 PMC7930837

[B46] Saporito L. Giammanco G. M. De Grazia S. Colomba C. (2013). Visceral Leishmaniasis: Host-Parasite Interactions and Clinical Presentation in the Immunocompetent and in the Immunocompromised Host. Int. J. Infect. Dis. 17 (8), e572–e576. doi: 10.1016/j.ijid.2012.12.024 23380419

[B47] Sasidharan S. Saudagar P. (2021). Leishmaniasis: Where Are We and Where Are We Heading? Parasitol Res. 120 (5), 1541–1554. doi: 10.1007/s00436-021-07139-2 33825036

[B48] Serafim T. D. Iniguez E. Oliveira F. (2020). Leishmania Infantum. Trends Parasitol 36 (1), 80–81. doi: 10.1016/j.pt.2019.10.006 31757772

[B49] Silveira F. T. Lainson R. Pereira E. A. de Souza A. A. Campos M. B. Chagas E. J.. (2009). A Longitudinal Study on the Transmission Dynamics of Human Leishmania (Leishmania) Infantum Chagasi Infection in Amazonian Brazil, With Special Reference to Its Prevalence and Incidence. Parasitol. Res. 104 (3), 559–567. doi: 10.1007/s00436-008-1230-y 18936975

[B50] Sohel M. A. (2016). Extracellular/Circulating MicroRNAs: Release Mechanisms, Functions and Challenges. Achievements Life Sci. 10, 175–186. doi: 10.1016/j.als.2016.11.007

[B51] Souza M. A. Ramos-Sanchez E. M. Muxel S. M. Lagos D. Reis L. C. Pereira V. R. A.. (2021). miR-548d-3p Alters Parasite Growth and Inflammation in Leishmania (Viannia) Braziliensis Infection. Front. Cell Infect. Microbiol. 11, 687647. doi: 10.3389/fcimb.2021.687647 34178725 PMC8224172

[B52] Takahashi R. U. Prieto-Vila M. Kohama I. Ochiya T. (2019). Development of miRNA-Based Therapeutic Approaches for Cancer Patients. Cancer Sci. 110 (4), 1140–1147. doi: 10.1111/cas.13965 30729639 PMC6447849

[B53] Tasew G. Gadisa E. Abera A. Chanyalew M. Abebe M. Howe R.. (2021). Whole Blood-Based *In Vitro* Culture Reveals Diminished Secretion of Pro-Inflammatory Cytokines and Chemokines in Visceral Leishmaniasis. Cytokine 145, 155246. doi: 10.1016/j.cyto.2020.155246 32828639

[B54] Tsuchiya S. Kobayashi Y. Goto Y. Okumura H. Nakae S. Konno T.. (1982). Induction of Maturation in Cultured Human Monocytic Leukemia Cells by a Phorbol Diester. Cancer Res. 42 (4), 1530–1536. 6949641

[B55] Tufekci K. U. Oner M. G. Meuwissen R. L. Genc S. (2014). The Role of microRNAs in Human Diseases. Methods Mol. Biol. 1107, 33–50. doi: 10.1007/978-1-62703-748-8_3 24272430

[B56] Varikuti S. Verma C. Holcomb E. Jha B. K. Viana A. Maryala R.. (2021). MicroRNA-21 Deficiency Promotes the Early Th1 Immune Response and Resistance Toward Visceral Leishmaniasis. J. Immunol. 207 (5), 1322–1332. doi: 10.4049/jimmunol.2001099 34341171

[B57] Vasquez R. E. Soong L. (2006). CXCL10/gamma Interferon-Inducible Protein 10-Mediated Protection Against Leishmania Amazonensis Infection in Mice. Infect. Immun. 74 (12), 6769–6777. doi: 10.1128/IAI.01073-06 16982826 PMC1698098

[B58] Vlachos I. S. Zagganas K. Paraskevopoulou M. D. Georgakilas G. Karagkouni D. Vergoulis T.. (2015). DIANA-Mirpath V3.0: Deciphering microRNA Function With Experimental Support. Nucleic Acids Res. 43 (W1), W460–W466. doi: 10.1093/nar/gkv403 25977294 PMC4489228

[B59] Wang Z. Wu X. Hou X. Zhao W. Yang C. Wan W.. (2020). miR-548b-3p Functions as a Tumor Suppressor in Lung Cancer. Lasers Med. Sci. 35 (4), 833–839. doi: 10.1007/s10103-019-02865-7 31485783

[B60] Wei F. Cao C. Xu X. Wang J. (2015). Diverse Functions of miR-373 in Cancer. J. Transl. Med. 13, 162. doi: 10.1186/s12967-015-0523-z 25990556 PMC4490662

[B61] WHO. (2021). World Health Organization. Available at: https://www.who.int/health-topics/leishmaniasis#tab=tab_1.

[B62] Xie Q. Wen H. Zhang Q. Zhou W. Lin X. Xie D.. (2017). Inhibiting PI3K-AKt Signaling Pathway Is Involved in Antitumor Effects of Ginsenoside Rg3 in Lung Cancer Cell. BioMed. Pharmacother. 85, 16–21. doi: 10.1016/j.biopha.2016.11.096 27930981

[B63] Xing T. J. Xu H. T. Yu W. Q. Wang B. Zhang J. (2014). MiRNA-548ah, A Potential Molecule Associated With Transition From Immune Tolerance to Immune Activation of Chronic Hepatitis B. Int. J. Mol. Sci. 15 (8), 14411–14426. doi: 10.3390/ijms150814411 25196343 PMC4159859

