# Corrigendum: miR-548d-3p Is Up-regulated in Human Visceral Leishmaniasis and Suppresses Parasite Growth in Macrophages

**DOI:** 10.3389/fcimb.2022.876035

**Published:** 2022-03-16

**Authors:** Eduardo Milton Ramos-Sanchez, Luiza Campos Reis, Marina de Assis Souza, Sandra Márcia Muxel, Kamila Reis Santos, Dimitris Lagos, Valéria Rêgo Alves Pereira, Maria Edileuza Felinto de Brito, Paul Martin Kaye, Lucile Maria Floeter-Winter, Hiro Goto

**Affiliations:** ^1^ Instituto de Medicina Tropical, Faculdade de Medicina, Universidade de São Paulo (IMTSP/USP), São Paulo, Brazil; ^2^ Departamento de Salud Publica, Facultad de Ciencias de La Salud, Universidad Nacional Toribio Rodriguez de Mendoza de Amazonas, Chachapoyas, Peru; ^3^ Graduate Program in Animal Science, Agrarian Sciences Center (CCA), Federal University of Paraiba (UFPB), Areia, Brazil; ^4^ Instituto de Ciências Biomédicas, Universidade de São Paulo, São Paulo, Brazil; ^5^ Veterinary Clinical Immunology Research Group, Departamento de Clínica Médica, Faculdade de Medicina Veterinária e Zootecnia, Universidade de São Paulo, São Paulo, Brazil; ^6^ York Biomedical Research Institute, Hull York Medical School, University of York, York, United Kingdom; ^7^ Instituto Aggeu Magalhães, Fundação Oswaldo Cruz (IAM/FIOCRUZ), Recife, Brazil; ^8^ Instituto de Biociências, Universidade de São Paulo, São Paulo, Brazil; ^9^ Departamento de Medicina Preventiva, Faculdade de Medicina, Universidade de São Paulo, São Paulo, Brazil

**Keywords:** *Leishmania (Leishmania) infantum*, microRNA, visceral leishmaniasis, THP-1 cells, pathogenesis, inflammation

In the original article, there is an error in the **Funding** section. The correct grant number for Medical Research Council is “(grants MR/P024661/1 and MR/S019472)”.

The authors apologize for this error and state that this does not change the scientific conclusions of the article in any way. The original article has been updated.

## Publisher’s Note

All claims expressed in this article are solely those of the authors and do not necessarily represent those of their affiliated organizations, or those of the publisher, the editors and the reviewers. Any product that may be evaluated in this article, or claim that may be made by its manufacturer, is not guaranteed or endorsed by the publisher.

