# Supplementary figures and images for: miR-548d-3p Is Up-Regulated in Human Visceral Leishmaniasis and Suppresses Parasite Growth in Macrophages
Source: Front Cell Infect Microbiol. 2022 Feb 10;12:826039. doi: 10.3389/fcimb.2022.826039 (PMC8900537; doi:10.3389/fcimb.2022.826039)

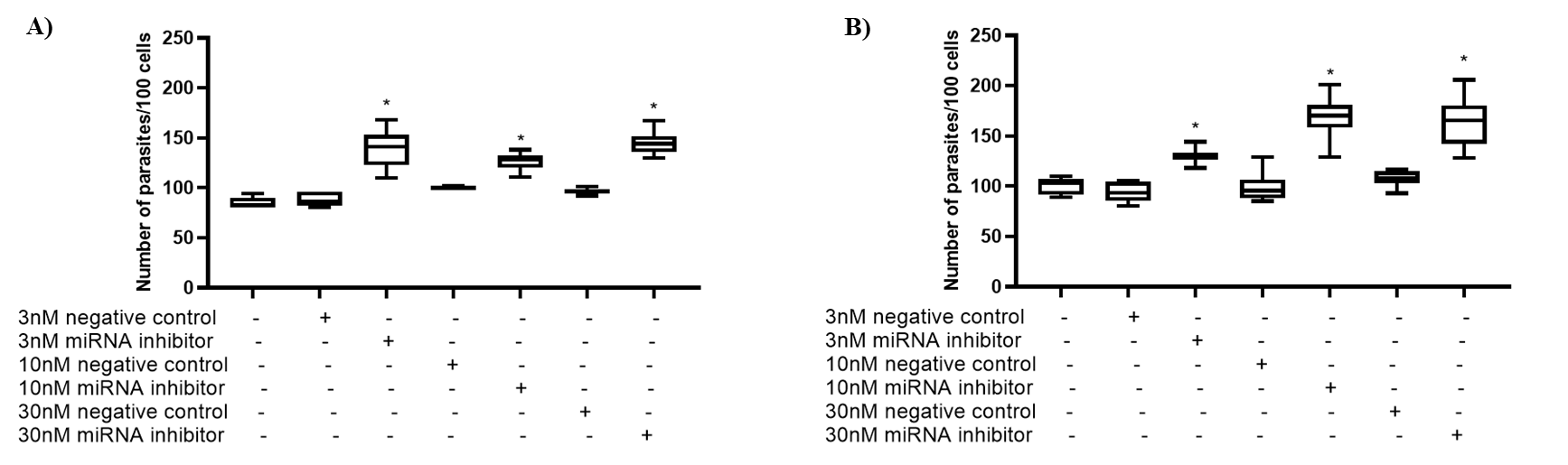

Supplement: Supplementary Figure 1 — Parasite load (number of parasites/100 cells) in L. (L.) infantum promastigote-infected THP-1 cells transiently transfected with miR-548d-3p inhibitor. THP-1 cells were transiently transfected with miR-548d-3p inhibitor (miR = 3, 10 and 30 nM) or negative control (NC - scrambled miRNA = 3, 10 and 30 nM) as described in Methods. After 24h, cells were infected with L. (L.) infantum promastigotes, and parasite load in macrophages was assessed at 6 hours post-infection (A) and 24 hours post-infection (B). * = P < 0.05 (one-way ANOVA and Tukey´s test) compared to the control groups. One representative experiment from three independent assays is shown. [file Image_1.tif]
