# Supplementary material for: miR-548d-3p Is Up-Regulated in Human Visceral Leishmaniasis and Suppresses Parasite Growth in Macrophages
Source: Front Cell Infect Microbiol. 2022 Feb 10;12:826039. doi: 10.3389/fcimb.2022.826039 (PMC8900537; doi:10.3389/fcimb.2022.826039)
Supplement: Supplementary file 2 [file DataSheet_1.pdf]

Table 1S. Predicted metabolic pathways and targeted predicted genes by modulated miRNAs.

PATHWAY	TARGETED GENES	GENE IDENTIFICATION
TGF-beta signaling pathway (hsa04350)	56	FST, TGFBR1, ID2, ROCK1, SMAD2, SMAD6, SMAD9, THBS1, PPP2CA, ACVR1B, SMURF2, BMPR1B, BMP5, PITX2, SMAD3, CUL1, INHBA, CDKN2B, LEFTY1, ID4, RHOA, ACVR1, SKP1, ACVR2B, ZFYVE16, DCN, SMAD4, E2F5, PPP2R1A, SMURF1, RBL1, SMAD5, ID1, ACVR2A, GDF6, BMP2, TFDP1, SP1, ACVR1C, TGFB2, EP300, BMPR1A, BAMBI, IFNG, LTBP1, ID3, SMAD7, NOG, MAPK1, CREBBP, PPP2R1B, LEFTY2, TGFBR2, BMP4, BMPR2, RPS6KB1
FoxO signaling pathway (hsa04068)	74	IRS2, BRAF, RBL2, STAT3, RAG1, TGFBR1, FBXO32, CCNB1, SOS2, SMAD2, NRAS, PRKAA2, STK4, SIRT1, PIK3CB, SETD7, KLF2, CCND2, SMAD3, CHUK, CAT, CDKN1B, IGF1R, EGFR, GADD45A, KRAS, HOMER2, ATM, NLK, G6PC, RAG2, PIK3R3, CCND1, SMAD4, S1PR1, SKP2, MAPK8, AKT1, GRM1, FOXG1, PIK3R1, SOS1, IRS1, INSR, PRKAA1, BCL6, PRKAB2, IGF1, TGFB2, CSNK1E, EP300, AKT3, SOD2, PRKAG2, PIK3CA, HOMER1, PRKAB1, FOXO3, USP7, ATG12, PTEN, SGK3, FOXO1, PLK4, MAPK1, CREBBP, IL10, CCNG2, TGFBR2, SGK1, MDM2, EGF, BCL2L11, C8orf44-SGK3
Wnt signaling pathway (hsa04310)	64	FZD7, CAMK2D, GSK3B, PRKCA, CSNK2A2, WNT16, FZD5, LRP6, TBL1X, BTRC, VANGL1, TCF7L2, WNT5A, CCND2, DKK2, ROCK2, PPP3R1, CTBP2, FRAT1, SMAD3, WNT2B, CUL1, WNT4, WNT3, LRP5, RHOA, FZD3, SKP1, PPP3CA, PRICKLE1, PLCB1, FZD4, SENP2, SOST, GPC4, JUN, CCND1, SMAD4, CTNNB1, AXIN2, NFATC2, MAPK8, CSNK1A1, VANGL2, CSNK2B, CSNK2A1, PRKX, RAC1, SIAH1, SFRP2, FZD1, PRKCB, PRICKLE2, CSNK1E, EP300, CXXC4, LEF1, WNT9B, NFATC3, DAAM1, MAP3K7, CREBBP, DVL2, TBL1XR1
HIF-1 signaling pathway (hsa04066)	55	CAMK2D, STAT3, ERBB2, MAP2K2, PIK3CB, CUL2, GAPDH, PIK3R5, ARNT, CDKN1B, EIF4EBP1, IGF1R, TLR4, VHL, HK2, RPS6KB2, AKT2, PDHA1, PLCG1, ENO1, PIK3R3, HIF1A, AKT1, TFRC, EGLN3, PDK1, PIK3R1, LDHA, INSR, PLCG2, EGLN2, EP300, AKT3, EIF4E2, PIK3CA, CDKN1A, SLC2A1, MKNK1, PFKFB2, EDN1, MKNK2, MTOR, ALDOA, PFKL, RELA, VEGFA, MAPK1, CREBBP, PDHB, PFKFB4, TEK, PGK1, EGLN1, PFKFB3, IL6R
